# Population structure and hybridisation in a population of Hawaiian feral chickens

**DOI:** 10.1038/s41437-022-00589-z

**Published:** 2023-02-01

**Authors:** Maria Luisa Martin Cerezo, Saioa López, Lucy van Dorp, Garrett Hellenthal, Martin Johnsson, Eben Gering, Rie Henriksen, Dominic Wright

**Affiliations:** 1 AVIAN Behavioural Genomics and Physiology group, IFM Biology, Department of Physics, Chemistry and Biology, Linköping University, Linköping, Sweden; 2 Wellcome Trust, 215 Euston Road, London, NW1 2BE UK; 3 UCL Genetics Institute, Department of Genetics, Evolution & Environment, University College London, Gower Street, London, WC1E 6BT UK; 4 SLU Uppsala, Department of Animal Breeding and Genetics, Swedish University of Agricultural Sciences, Uppsala, Sweden; 5 Department of Integrative Biology and Program in Ecology, Evolution and Behavior, Michigan State University, East Lansing, MI USA; 6 Department of Biological Sciences, Halmos College of Natural Sciences and Oceanography, Nova Southeastern University, Fort Lauderdale, FL USA

**Keywords:** Genetic variation, Evolutionary biology

## Abstract

Chickens are believed to have inhabited the Hawaiian island of Kauai since the first human migrations around 1200AD, but numbers have peaked since the tropical storms Iniki and Iwa in the 1980s and 1990s that destroyed almost all the chicken coops on the island and released large numbers of domestic chickens into the wild. Previous studies have shown these now feral chickens are an admixed population between Red Junglefowl (RJF) and domestic chickens. Here, using genetic haplotypic data, we estimate the time of the admixture event between the feral population on the island and the RJF to 1981 (1976–1995), coinciding with the timings of storm Iwa and Iniki. Analysis of genetic structure reveals a greater similarity between individuals inhabiting the northern and western part of the island to RJF than individuals from the eastern part of the island. These results point to the possibility of introgression events between feral chickens and the wild chickens in areas surrounding the Koke’e State Park and the Alaka’i plateau, posited as two of the major RJF reservoirs in the island. Furthermore, we have inferred haplotype blocks from pooled data to determine the most plausible source of the feral population. We identify a clear contribution from RJF and layer chickens of the White Leghorn (WL) breed. This work provides independent confirmation of the traditional hypothesis surrounding the origin of the feral populations and draws attention to the possibility of introgression of domestic alleles into the wild reservoir.

## Introduction

Feralisation occurs when a domestic population escapes the controlled environment where they lived and return to the wild (Henriksen et al. 2018). Feralisation entails an exposure to new pressures, such as predation and natural and sexual selection, which were absent in the domestic environment (Gering et al. 2019; Henriksen et al. 2018). Unlike domestic populations, whose ancestors are generally not available, feral populations start from a well defined domestic ancestor, both genetically and phenotypically, thus providing a unique opportunity to study how the genome responds to selective pressure. While endoferal populations, originated from a single domestic group, are the simplest model to elucidate the processes occurring when domestic populations are exposed to natural and sexual selection, exoferal populations, originated from the mixing of multiple domestic lineages, hybrid lineages, or between domestic and wild lineages, can be useful to further understand the effects of introgression and admixture in feral populations (Gering et al. 2019).

The feralisation process has occurred worldwide in a wide range of organisms, including mammals (dogs (Zhang et al. 2020), cats (Doherty et al. 2017), horses (Scorolli and Cazorla 2010), pigs (Petrelli et al. 2021), goats (Cowan et al. 2020)), birds (chickens, (Johnsson et al. 2016), pigeons (Carlen and Munshi‐South 2021), ducks (Lavretsky et al. 2020)), fish (salmon (Wringe et al. 2018), guppies (Swaney et al. 2015), cichlids (Singh et al. 2014)), insects (honeybee (Kohl and Rutschmann 2018)) and plants (rice (Zhang et al. 2018), rye (Burger et al. 2007), and radish (Pandolfo et al. 2016). Feral populations can impact native species in multiple aspects, by competing for the same resources, such as food or shelter, and by increasing predation, the transmission of diseases and the degradation of the environment. Furthermore, exoferal populations coexisting with their wild counterparts, can generate important conservation issues, due to the potential introgression of domestic alleles into the wild population. In order to properly manage feral populations, it is necessary to know how and when these populations have originated and to what extent hybridisation has or has not occurred. This information can be crucial to determine the best method of population control and inform how best to avoid or limit such occurrences in the future.

On the island of Kauai, in the Hawaiian archipelago, chickens first arrived in AD 1200, when the Polynesian settlers brought Red Junglefowl (RJF), the wild-type chicken, with them (Kirch 2011; Thomson et al. 2014). The second known arrival of RJF onto the island occurred in 1939 when 857 Pacific RJF were intentionally released into the wild, to maintain the naturalised population, which suffered a large reduction in their numbers because of the increased hunting and predation pressure introduced by the European settlers (Pyle and Pyle 2017). To this day, chickens in Koke’e State Park are referred to as Moa and are considered to be descended from the RJF that were originally brought over by the Polynesian settlers (Denny 1999; as cited in Pyle and Pyle 2017). These are believed to be the reservoir population for the RJF alleles that are now seen in the feral populations on the island, though direct genetic sampling of these birds is lacking (Gering et al. 2015). Domesticated chickens first arrived in Hawaii at the end of the 18th century (Caum 1933; as cited in Pyle and Pyle 2017), though their numbers increased markedly as human habitation increased on the island. Hatcheries have been present on the island, with at least one company selling chicks since 1935 (http://www.asagihatchery.com). During disruptions caused by the tropical hurricanes Iwa (in 1982) and Iniki (in 1992) almost all the domestic chickens escaped into the wild (Johnsson et al. 2016). Domesticated chickens have, since then, roamed freely on the island, becoming a large feral population. Prior to these storms, the numbers of feral birds (either from escaped domestic birds or from one of the introduced RJF populations) were low, but subsequently, numbers have risen markedly (Gering et al. 2015). Using a combination of mitochondrial analyses, plumage analysis and vocalisation analysis, Gering et al. 2015 found the feral birds present on the island of Kauai to be an admixed population with origins in both RJF and domestic chickens (Gering et al. 2015). The Polynesian RJF mitotype (Clade D) was found to be present in the feral population, as well as the domestic mitotype (Clade E). RJF birds have a distinct vocalisation that separates them from domestic chickens, a truncated last syllable of the male call as compared to a longer last syllable in domestic males. The feral birds in Kauai were also found to have a wide variation in calls, with males with more RJF appearance also having a more RJF call and vice-versa. Finally, despite birds possessing generally RJF plumage appearance, introgression with white and brown feathers were also common (Gering et al. 2015).

It is not known which domestic breeds were released during the hurricanes, and therefore which have contributed to the feral chickens today, though the largest hatchery on the island produces Cornish Rock, White Leghorns and brown Layer chickens, with these standard Layer breeds thus potentially the most likely origin. However, currently there is no information about the breeds that contributed to establishing this feral population, nor any genetic information as to when and to what extent hybridisation between RJF and domestic birds occurred. Genomic data have long been used to link abiotic events to population divergence (Knowlton and Weigt 1998; Qin et al. 2013). Nevertheless, classical methods rely on phylogenetic sorting and are therefore unsuitable for testing how recent (e.g. anthropogenically-driven) environmental changes impact the evolution of contemporary gene pools.

More generally, hybridisation and introgression can aid in contributing adaptive variation to a species or population (Barton 2001), and thus can be an important process to quantify. In domestication, hybridisation has been prevalent in numerous domestic species (Arnold 2004; Götherström et al. 2005; Miller and Gross 2011), as well as during feralisation (exoferal introgressions in wild populations being common (Gering et al. 2019)). For example, Grant 1981 first posited that clusters of linked genes in plants were as a result of repeated hybridisations with different populations containing desirable traits. Such a pattern of linkage in domestic QTL hotspots also appears to exist in the chicken (Wright et al. 2010; Johnsson et al. 2014; Wright 2015), suggesting that such a mechanism is also operating in the domestic chicken. During chicken domestication, introgressions from other species and subspecies have also been identified. Grey Junglefowl was initially demonstrated to be introgressed into the domestic genome (Eriksson et al. 2008), whilst more recently introgressions from Ceylon and Green Junglefowl (Lawal et al. 2020) and *G.g. Spadiceus* (Wang et al. 2020) have also been found. The reverse has also occurred, with domestic introgressions occurring in native RJF populations in Singapore (Wu et al. 2020).

Here we leverage advanced tools (chromoPainter, fineSTRUCTURE and GLOBETROTTER) for local ancestry assignment, admixture detection and dating to examine the hypothesis that disruption caused by recent hurricanes drove hybridisation of domestic and wild gene pools, and facilitated adaptation in Kauai’s feral chickens. By comparing haplotype sharing patterns observed between chickens sampled on three different geographic regions within the island of Kauai, we have estimated the date when this hybridisation event occurred. We then tally the derived date with the known timing of hurricanes Iwa (1982) and Iniki (1992), to ascertain the timing and extent of the hybridisation with the genetic data. In this study, we determined the relationship between sampled individuals across the island using a model-based Bayesian clustering method. We then compare these genetic clusters and determine evidence for any potential admixture event(s) that may have occurred between them. Finally, by combining our dataset with a previously published dataset of some of the most common commercial chicken breeds, we have determined the most probable domestic source of the current feral population. This information may be of particular relevance to the correct management and conservation of the wild population of RJF on the island of Kauai.

## Methods

### Sample collection and sequencing

**Fig. 1:**
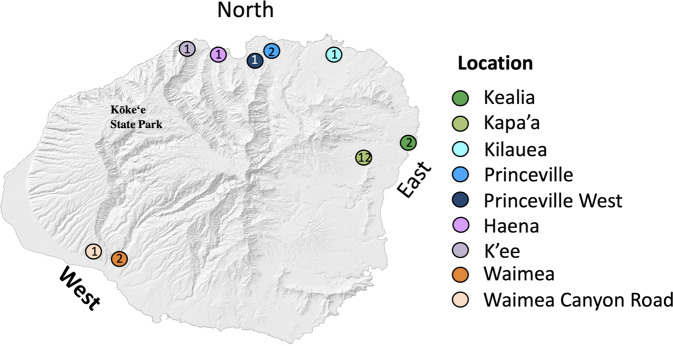
Sampling localities on the island of Kauai for the 23 feral Gallus gallus. Each colour provides a different location. The numbers inside the circles indicate the number of samples from each location. Image modified from Gering et al. (2015).

A total of 23 feral chicken samples donated by private individuals from the island of Kauai, in Hawaii in 2013 and imported to Sweden under permit DRN 6.2.18-1361/13, were used for this study (Gering et al. 2015; Johnsson et al. 2016) (Fig. 1), as well as one RJF from the zoo population of Götala Research Station, maintained in our facility in Sweden (ugc_610), which represents the wild type. Each of these 23 samples was individually sequenced.

Samples were collected from nine different regions on the island (see Supplementary Table 1), located either on the north, east or west side of the Island. DNA was extracted from blood samples preserved in RNA later following a salt extraction protocol (Aljanabi 1997) and sequenced using a SOLID 5500xl platform at Uppsala Genome Center, part of the National Genomics Infrastructure. Bioinformatic analyses were performed using computational resources provided by the Uppsala Multidisciplinary Center for Advanced Computational Science (Lampa et al. 2013). 75bp sequences were aligned to the chicken reference genome Galgal4 with LifeScope Genomic Analysis Software version 2.5.1 (Life Technologies), producing an average genome coverage of X5 per individual. SNPs were called following Johnsson et al. (2016). Briefly, duplicates were removed with Markduplicates from Picard version 1.92 (http://broadinstitute.github.io/picard/), sequences were realigned around indels with IndelRealigner from GATK and quality scores were recalibrated with BaseRecalibrator from GATK (DePristo et al. 2011).

### Genetic structure and ancestry

The genetic structure of Kauaian chickens sampled on Kauai, including RJF, was visualised through Principal Component Analysis (PCA) implemented in Plink version 1.9 (https://www.cog-genomics.org/plink/1.9/) (Purcell et al. 2007). In order to analyse patterns of haplotype sharing between RJF and feral chickens, we used ChromoPainter (Lawson et al. 2012), a more powerful approach than PCA and Admixture/STRUCTURE analysis due to the explicit consideration of linkage disequilibrium (Leslie et al. 2015; Van Dorp et al. 2018). ChromoPainter identifies, at each position of the genome, which donor sample has a haplotype pattern that best matches the patterns observed in the recipient sample. The probability of matching then indicates which recipient is most closely related to a particular donor over all the possible donors considered in the analysis so that each genome is considered as a mosaic of all others excluding self-copying (the matching of a recipient to itself in the donor panel). In this way, we are able to construct the feral population as a set of domestic and wild (RJF-like) haplotypes which are explicitly compared to each other. These haplotype blocks, also called chunks, are fragments of continuous DNA that are shared between the recipient and each one of the feral and RJF donors and coancestry matrices are constructed by considering these pairwise patterns of matching genome-wide.

First, genotype data were phased with SHAPEIT (Delaneau et al. 2012) using the sex-averaged recombination map for chicken populations published by Elferink et al. (2010) and following the protocol described in Johnsson et al. (2016). Population size (Ne) and mutation rate (Mut) were subsequently estimated from the phased data by running ChromoPainter’s expectation maximisation algorithm on all the individuals and chromosomes. The average values, weighted by chromosome size, obtained were Ne = 3166 and Mut = 0.0287574 which were provided as fixed values (*-n* and *-M* flags) in a final ChromoPainter run.

### Haplotype-based clustering analysis and admixture dating

The coancestry matrix produced by ChromoPainter, provides the number (count) of haplotypic chunks shared between all pairs of individuals in the datasets. This matrix was provided to fineSTRUCTURE to classify individuals from different regions of Kauai into homogeneous groups based on their shared ancestry. fineSTRUCTURE defined a set of eight clusters, or groups. Weighted Fst values between feral groups and the RJF were calculated with Plink v1.9 using the Weir and Cockerham method (Weir and Cockerham 1984). Each one of the fineSTRUCTURE defined groups was then analysed for evidence of admixture in GLOBETROTTER (Hellenthal et al. 2014) using the RJF and the other groups of feral chickens as surrogates. GLOBETROTTER analyses patterns of linkage disequilibrium to describe and date admixture events that have occurred in the last 150 generations. This approach uses Chromopainter-ascribed local ancestry patterns to resolve admixture signals using different specifications of potential surrogates for the admixing sources. As GLOBETROTTER facilitates testing of different surrogate sets for putative admixing sources, this can allow inference of admixture events at more recent and more ancient timescales. For example, in our dataset, when recent admixture events were detected, dominated by the signal of mixing of closely related feral surrogates, they were removed from the possible surrogate panel in order to explore whether these recent signals were masking older events. GLOBETROTTER analyses were run using ‘Nullind0’ and ‘Nullind1’ options as recommended in Hellenthal et al. (2014). Specification of Nullind0 supports the use of target clusters with a single individual, allowing RJF to be used a target, while Nullind1, which requires at least two individuals in the target cluster, can account for unusual patterns of linkage disequilibrium. These patterns can, otherwise, be confounded by admixture, making the Nullind1 approach more accurate when greater than one sample is present in the target group. GLOBETROTTER estimates admixture events scaled by the number of generations since the initial mixing occurred. Based on the time needed to reach sexual maturity and our personal observation of multiple breeding seasons occurring throughout the year on the island, we consider a generation time of around 6 months.

### Domestic contribution

To determine the contribution of domestic and RJF chickens to the feral island population, ChromoPainter was run using the single RJF sample as a recipient and different specifications of domestic breeds and RJF as surrogates for the admixing source. Samples from Rubin et al. (2010) were selected to encompass the diversity of sampled domestic breeds and also a wider diversity of RJF. Shortly, these data were pooled samples that included four layers of lines (a selected line developed at the Swedish University of Agricultural Sciences), White Leghorn line 13 (WLA), commercial White Leghorn (WLB), an obese strain derived from White Leghorns in 1955 (OS) and commercial Rhode Island Red (RIR)), four broiler lines (two commercial broiler, CB1 and CB2, and low (BL) and high (BH) growth selection lines established from White Plymouth Rock chickens in 1957) and eight RJF males from two different zoo populations. These samples were pool-sequenced and their haplotypes were reconstructed for each pooled strain. Full details of the pooling process can be found in Rubin et al. (2010).

### Haplotype phasing and reconstruction from pooled data

Kauaian chickens' sequences were phased jointly using SHAPEIT (Delaneu et al. 2012) and incorporating the sex-averaged recombination map for all chicken populations first published in Elferink et al. (2010). Prior to phasing, data were pruned to remove triallelic SNPs, sites with a rate of missing data >0.05 and SNPs with a minor allele frequency <0.01 using PLINK v1.07 (Purcell et al. 2007), leaving a total of 3,745,889 markers. In the case of the pooled domestics and pooled RJF from Rubin et al. (2010), in order to build the haplotyes required for ChromoPainter analyses, we followed the protocol set out in Gering et al. (2015) which was shown to reliably recover population diversity and selective sweeps in RJF and domestic breeds. In particular, we sampled alleles at each SNP based on the pooled data read probabilities and generated a haplotype for each strain. In cases where a read probability was not given we sampled each allele type with 50% probability.

## Results

### Genetic structure

Principal Component Analysis (PCA) identifies clear genetic differentiation amongst samples collected from the three different locations (north, east, or west side of the island) sampled on the island of Kauai and also with the RJF sample (Supplementary Figure 1). The observed genetic structure reflects the geographical distribution of the samples, with individuals from Eastern Kauai distributed all the way along PC component one and samples from the Western and Northern groups clustering more closely to one another. Component two showed a clear differentiation between feral chickens, on the lower area of the graph, and the RJF, on the upper part, indicating genetic differentiation between feral chickens and RJF. Furthermore, PCA indicated some degree of differentiation between feral chickens from Western Kauai and the rest of the Kauaian samples, though this was not apparent for all of the Western Kauai individuals.

**Fig. 2:**
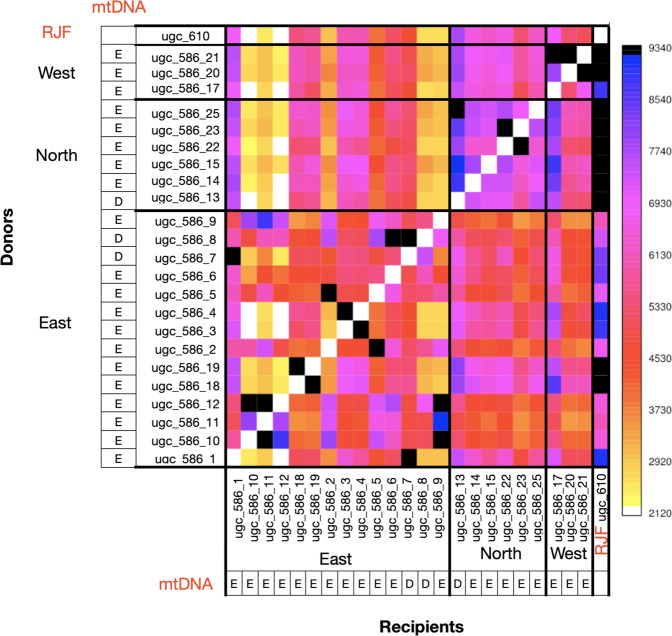
ChromoPainter coancestry matrix providing the number of haplotype segments (chunk counts) shared for each recipient chicken (*x*-axis) while using all the other chickens as donors (*y* axis). Colours provide the number of haplotype segments shared amongst individuals, with darker colours indicating a stronger similarity between samples. Individuals are ordered by sampling region with black lines separating each population. Mitochondrial haplogroups (Gering et al. 2015) for each sample are indicated immediately before the sample name.

Ancestry analysis (Fig. 2) identified a clear differentiation between RJF and feral chickens, with RJF sharing more haplotype segments with feral chicken from the north and west of Kauai than with the eastern population. This pattern agrees with the results obtained via PCA (Supplementary Fig. 1), where the first component separates most of the Eastern Kauaian individuals from the Northern and Western populations and RJF. We also observed that chickens from the same region share, in general, more haplotype segments between them than with chickens from other geographical regions, indicating population differentiation between the different locations sampled. Furthermore, haplotype-based analyses suggest a closer relationship between the Northern and Western populations than with the Eastern population (Fig. 2).

### Haplotype-based structure analysis

**Fig. 3:**
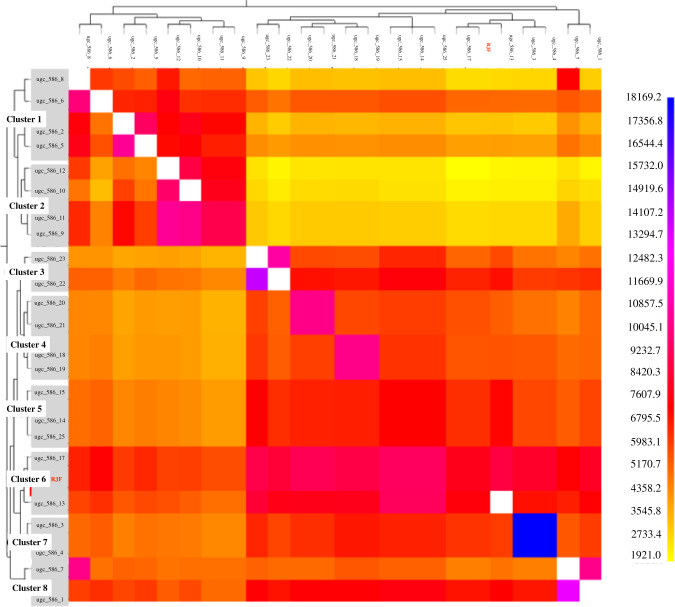
fineSTRUCTURE coancestry matrix with the order of donor and recipient samples based on the eight unique clusters identified. Colours provide the shared chunk counts, with darker colours suggesting a closer relationship or similarity between samples. Donors are shown on the y-axis while recipients are shown on the *x*-axis).

fineSTRUCTURE analysis identified eight different feral clusters that were in general agreement with the geographical origin of the samples (Fig. 3). Clusters 1, 2, 7 and 8 correspond to samples from Eastern Kauai, while clusters 3 and 5 include samples from Northern Kauai. However, clusters 4 and 6 include Kauaian samples from multiple locations, cluster 4 is formed by samples from the western and eastern populations, while cluster 6 includes samples from the north and the west of the island and also the RJF.

The strongest differentiation was found between cluster 2 and the RJF (Fst =0.4480) (Supplementary Table 2). In contrast, the smallest genetic differences were found between the clusters including individuals from multiple locations (clusters 4 and 6) and cluster 5 (consisting of individuals from the north of the island). Fst values, in this case, ranged from 0.0060 (cluster 5:cluster 6) and 0.0193 (cluster:cluster5) to 0.0218 (cluster 4: cluster 6).

### Admixture events

GLOBETROTTER identified, in the majority of cases, evidence of admixture occurring between sources best represented by feral clusters between seven to 44 generations ago. This suggests that mixing between feral clusters is common. An exception was Cluster 3, with a 50% contribution from RJF and a 50% contribution from another feral cluster, Cluster 6. This admixture event was dated to 37 generations before sampling (31.69–44.36). Considering a six months generation time, as previously explained, this allows calibration of the inferred admixture event to ~19 years (15.84–22.19 95% CI) before sampling. We note that no admixture event could be detected in Cluster 2, suggesting that Cluster 2 may be more isolated than the other clusters from the eastern region. Results specifying Nullind0 and Nullind1 options were broadly consistent between them (Table 1 and Supplementary Table 3).

**Table 1:** Inferred date of admixture calculated by GLOBETROTTER with Nullind1 specification.

Target	Gen.1date	Gen.1date.boots	Prop.source1	Best.source1	Prop.source2	Best.source2
Cluster1	9.63	(7.43–12.19)	0.57	Cluster2	0.43	Cluster3
Cluster2	No admixture signal detected					
Cluster3	37.06	(31.69–44.36)	0.50	RJF	0.50	Cluster6
Cluster4	44.59	(34.66–54.80)	0.50	Cluster5	0.50	Cluster6
Cluster5	35.50	(26.75–45.68)	0.79	Cluster3	0.21	Cluster2
Cluster6	28.64	(23.05–34.41)	0.81	Cluster3	0.19	Cluster2
Cluster7	38.18	(21.52–63.18)	0.86	Cluster4	0.14	Cluster1
Cluster8	7.06	(4.11–9.12)	0.76	Cluster4	0.24	Cluster2
RJF	42.25	(42.25–42.25)	0.88	Cluster4	0.12	Cluster2

The date of the admixture event for each cluster (Target) is given in generations (Gen.1date) along with the 95% confidence interval for the number of generations calculated by bootstrapping over 100 iterations of the GLOBETROTTER algorithm (Gen.1date.boots). The admixing proportion and the best surrogate for the detected admixture event (Prop.source1, Best.source1 and Prop.source2, Best.source2) are provided. Source 1 is the major contributor while source 2 is the minor contributor. No admixture event was detected in Cluster 2. Results for RJF are provided without standardising by a null individual (nullind 0) given the target group contains a single recipient sample.

We note in some cases we identify fairly recent admixture events (7–9 generations ago) which likely reflect recent mixing between feral populations on the island and may mask older events which have contributed to the diversity of haplotypes recovered. In particular we reperformed admixture analysis Cluster 1 samples excluding Cluster 2 and Cluster 8 as possible surrogates for the admixing sources. The inferred admixture event was subsequently dated to 67.79 generations with a 50% contribution from a source best modelled by Cluster 5, a feral cluster, and 50% from a source best modelled by the RJF (Supplementary Fig. 2). Assuming a generation time of six months, this admixture can be dated to ~1981 (1976–1995).

### Domestic contribution

**Fig. 4:**
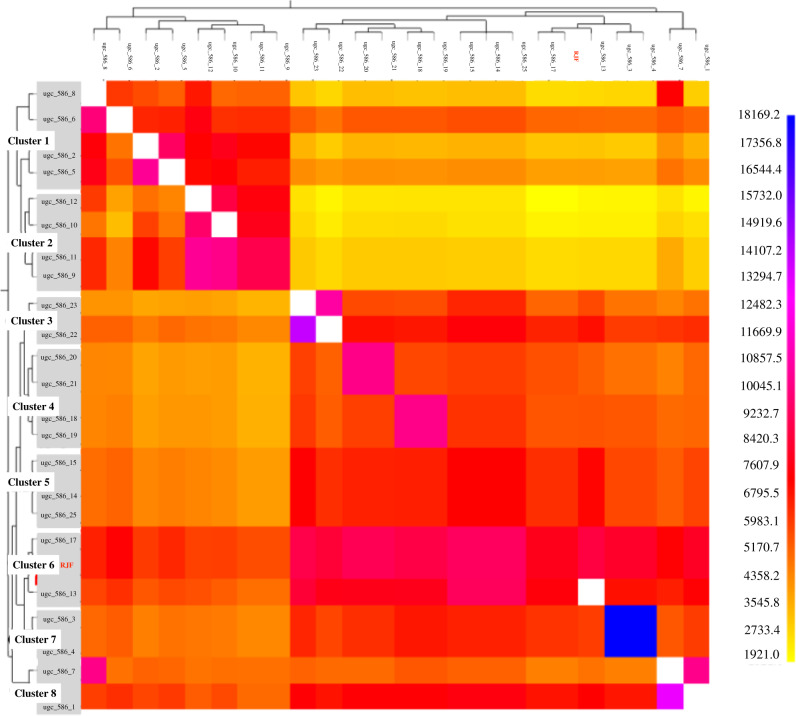
ChromoPainter coancestry matrix providing the haplotype sharing pattern for each recipient chicken (*x*-axis) while using pooled data reconstructed from domestic and wild data as donors (*y* axis). The colour scale provides the number of haplotype segments shared amongst individuals, with darker colours indicating a stronger similarity between samples. Individuals on the x-axis are ordered by sampling location. The *x* axis displays individual IDs while the y-axis displays the ID for each of the pools used: a commercial White Leghorn (WLB, *n* = 8), two broiler growth selection lines established from White Plymouth Rock chickens in 1957, a low growth (BL, *n* = 11) and a high growth one (BH, *n* = 11), RJF males from two different zoo populations (RJF, *n* = 8), commercial Rhode Island Red (RIR, *n* = 8), an obese strain derived from White Leghorns in 1955 (OS, *n* = 10), a selected line developed at the Swedish University of Agricultural Sciences, also known as White Leghorn line 13 (WLA, *n* = 11) and two commercial broilers (CB1, *n* = 10 and CB2, *n* = 10).

Patterns of haplotype sharing inferred by ChromoPainter when assessing feral chickens from Kauai are provided in Fig. 4. Relative to other donors in the dataset we found that feral chickens share most haplotype blocks with both RJF and layer domestic chickens (from the obese strain (OS)). The mean percentage of chunks assigned to the OS Layer was 12% (0.11–0.12) and the mean percentage of chunks assigned to the RJF was also 12% (0.11–0.13). This haplotype-sharing pattern is consistent with an admixture event between the naturalised population of RJF in the island of Kauai and layer domestic chickens. Similarly, more haplotype blocks were also shared between feral chickens and layers WLA chickens, making the layer WLA population another suitable candidate for the domestic source of this feral population. Both domestic candidates are derived from the same breed of layers, the White Leghorn. The RJF used for our study shared haplotype blocks mainly with RJF from Rubin et al. (2010), indicating the robustness of the previous analysis and that Red Junglefowl represent a clearly genetically distinct group.

## Discussion

By comparing patterns of haplotype sharing both within a recent feral population of chickens from the island of Kauai and between this feral population and domestic and RJF populations, we have been able to infer the time of the initial admixture event and also determine the most plausible domestic source of this feral population. The analyses performed in this study provide genetic evidence of at least one admixture event between feral chickens and RJF on the island of Kauai occurring around the year 1981. This timeframe concurs with hurricane Iwa (which occurred in 1982), which destroyed large parts of the island, releasing domestic chickens into the wild. Our assessment of this population, jointly with different domestic breeds, indicates that the extant feral population shares more haplotype blocks with layers from the White Leghorn breed (Rubin et al. 2010). However, most of the feral individuals analysed shared more haplotype blocks with RJF than with any of the domestic breeds. This haplotype-sharing pattern could be consistent with an admixture event between White Leghorns, one of the breeds sold by Asagi hatchery (one of the oldest companies in the Hawaiian Islands), and the naturalised population of RJF. The contribution of the four broiler breeds was lower than for any of the strains derived from White Leghorns, which is in line with expectations, considering the difficulties that broilers have in reproducing even in domestic conditions. Broiler chickens, selected for accelerated growth, can develop skeletal and cardiovascular problems that increase their mortality rate and reduce their reproductive capacity (Hartcher and Lum 2020). The extreme artificial selection to which broilers have been subjected may have made them maladapted or unfit to survive and reproduce in a natural environment, reducing their possibilities of feral establishment.

The mitochondrial haplogroup composition of this feral population (Gering et al. 2015) has previously identified the coexistence of two groups: the D haplogroup, mainly restricted to Asia and the Pacific area, representing the wild-type chickens, and probably introduced by the Polynesian settlers, and the E haplogroup, found mainly in modern European chicken breeds (Gering et al. 2015). Gering et al. (2015) revealed that 20 out of 23 individuals belong to haplogroup E, which most likely indicates a domestic origin. Interestingly, one of three individuals (ugc_586_7) with the D haplogroup, the wild type haplogroup, shares more haplotype blocks with the layer domestic chickens than with RJF, while the two other individuals (ugc_586_8 and ugc_586_13) follow the general trend and share a similar number of haplotype blocks with the two potential founders, RJF and OS, (ugc_586_8) or a higher number of haplotype blocks with RJF than with any domestic breed (ugc_586_13). Due to the characteristics of the mitochondrial genome, lack of recombination, and maternal inheritance (Alexander et al. 2015), a single cross with RJF could have introduced the D haplogroup into mainly domestic chickens. The reverse of this phenomenon can also occur when individuals that share more haplotype blocks with RJF than to any domestic breed could carry a domestic mitochondrial haplogroup (e.g. ugc_586_17).

Admixture analysis with whole-genome data has previously demonstrated that the population of Kauai is an admixed population between domestic chickens and naturalised RJF brought to the island at different times (Gering et al. 2015). However, until now, the timing of this admixture event was unknown as well as what potential domestic sources comprised the hybrid population. Population structure analysis has shown that most of the individuals cluster with individuals from the same geographic region. This appears to represent general site fidelity amongst these populations, though given the small size of the island (approximately 90 miles diameter) and the fact that these are birds capable of flight, it is expected that admixture between geographically grouped genetic clusters can occur. Only two exceptions were found: cluster 4 which contains samples from the east and west of the island and cluster 6 which contains the RJF and samples from the west and the north of the Island. While the grouping observed in cluster 4 cannot be explained by geography, the two samples that cluster with the RJF were captured in the areas bordering the Koke’e State Park and the Alaka’i plateau, an area of difficult access which has long been considered as the major reservoir of RJF’s on the island (Pyle and Pyle 2017). The proximity to this region makes these two populations more likely to admix with RJF and increases the risk of introgression of domestic alleles into the RJF reservoir. From a conservation point of view, it will be crucial to analyse the population inhabiting the Alaka’i plateau and the Koke’e State Park to determine if domestic alleles have already been introduced into the population and clarify if there is still a reservoir of wild RJF in the area. Our results do indicate that the individuals that are closest to the RJF live in close proximity to this area. If introgression of domestic alleles has yet to occur, preventive measurements should be taken to preserve this genetic reservoir. However, if introgression of domestic alleles into this reservoir of RJF has already occurred, feral populations could act as a reservoir itself of genetic variability for domestic chickens.

The ChromoPainter/fineSTRUCTURE/GLOBETROTTER pipeline is a suite of haplotype-based methods which have been shown to be able to resolve population differences more efficiently than methods treating markers independently, revealing more subtle population patterns, than would otherwise be detectable (Lawson et al. 2012; Leslie et al. 2015; Van Dorp et al. 2018). However, there are some important caveats in our analysis that should be taken into account. Here, we have been able to estimate the time of admixture between the naturalised population of RJF and domestic chickens, now feral, on the island of Kauai. Other admixture events, between feral groups and with RJF, have also been detected. All of the recovered events are dated as fairly recent, indicating multiple contacts have occurred between groups living in different areas of the island despite geographic separation. It is therefore possible that more (unsampled) feral groups could have interbred with the naturalised population of RJF during the history of this population. In addition, as feral groups are themselves a composite of wild and domestic alleles, and some of the admixture events between feral clusters could also signpost to recent admixture events with RJF. In this analysis we elect to use feral clusters as surrogates given they are the best representation of the domestic chickens (original founding haplotypes) that formed the feral population of Kauai today. Secondly, the ChromoPainter/fineSTRUCTURE/GLOBETROTTER pipeline is haplotype-based and was not originally designed to be used with pool data. More typically, haplotype blocks should be inferred individually while here, we have reconstructed domestic and haplotype blocks by using the pooled probabilities from each SNP. However, we anticipate that our allele sampling protocol adequately captures the dominant diversity in the sample as also supported by the strong patterns of population structure we recover. As the pooled data was only used in the domestic contribution analysis (i.e. the calculation of the most likely domestic breeds to derive the feral birds), this was not an issue for dating the hybiridsation event in any case. Nevertheless, this caveat should be born in mind when considering the domestic donors for the feral population.

In summary, our haplotype-based analyses have shed light on the origin of the feral population of Kauai, dating for the first time the admixture event that has occurred in the island of Kauai, with only a deviation of one year between the tropical storm that released the domestic chickens into the wild and our point estimation. We can thus corroborate the archaeological/anthropological knowledge of the histories of these chickens using independent genetic data. We have also analysed the genetic composition of this population, giving an approximation to the domestic origin of these birds, a component that appears to be closely related to White Leghorn layers. Our work demonstrates that admixture between different feral groups and with the RJF on the island has been occurring during the last 40 years. These results again emphasise the challenge of conservation management of chicken populations on the island, in particular in terms of whether to protect or eradicate certain groups given extensive admixture between them.

## Supplementary information

Supplementary material

## Data Availability

All the data used for this study was previously published. Sequenced data is available at NCBI (SRA accession numbers SRP052017 and SRP001870). Accession number of each one of the samples can be found in Supplementary Table 1.
