# Supplementary material for: Population structure and hybridisation in a population of Hawaiian feral chickens
Source: Heredity (Edinb). 2023 Feb 1;130(3):154–62. doi: 10.1038/s41437-022-00589-z (PMC9981564; doi:10.1038/s41437-022-00589-z)
Supplement: Supplementary file 1 — Supplementary material [file 41437_2022_589_MOESM1_ESM.doc]

**Supplementary files.**

**Contents: Supplementary Figures and Tables for Martin Cerezo et al. “Population Structure and Hybridisation in a Population of Hawaiian Feral Chickens”.**


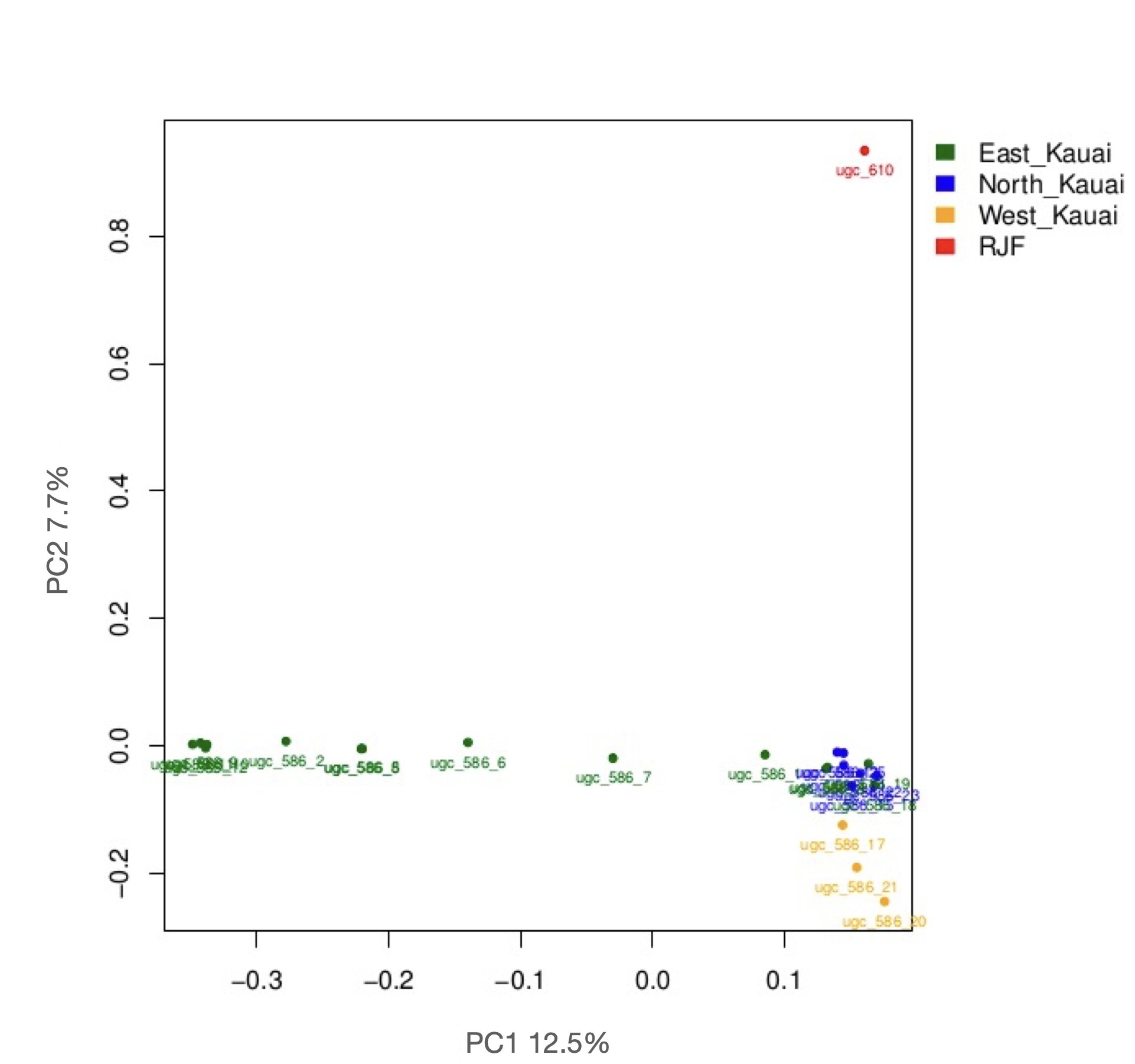


Supplementary Figure 1:Principal Component Analysis (PCA) performed on the genetic data using Plink v1. Samples are coloured according to the region of origin. IDs for each sample are also printed. PC1 explained 12.5% of the total variation, PC2 explained 7.7% of the total variation.

*Supplementary Figure 2: LD decay curves (in cM) for Cluster 1 when excluding Cluster 2 and Cluster 8 as possible surrogates. Coancestry curves (black line) show the probability of coping two haplotypic segments from RJF and/or cluster 5. Different admixing sources can be inferred from the positive slope observed for the RJF-cluster 5 comparisons (bottom).*

*Supplementary Table 1: Accession number, sample ids, localities, population group and mtDNA haplogroup for each one of the samples used in this study (Gering et al., 2015).*

| *Accesion number* | *Sample_id* | *Origin* | *Locality* | *Group* | *mtDNA haplogroup* |
| --- | --- | --- | --- | --- | --- |
| SRR1770445 | ugc_586_1 | Feral | Kapa'a#1 | East | E |
| SRR1772149 | ugc_586_2 | Feral | Kapa'a#2 | East | E |
| SRR1772170 | ugc_586_3 | Feral | Kapa'a#3 | East | E |
| SRR1772172 | ugc_586_4 | Feral | Kapa'a#4 | East | E |
| SRR1772175 | ugc_586_5 | Feral | Kapa'a#6 | East | E |
| SRR1772178 | ugc_586_6 | Feral | Kapa'a#7 | East | E |
| SRR1772194 | ugc_586_7 | Feral | Kapa'a#8 | East | D |
| SRR1772198 | ugc_586_8 | Feral | Kapa'a#9 | East | D |
| SRR1772199 | ugc_586_9 | Feral | Kapa'a#11 | East | E |
| SRR1772215 | ugc_586_10 | Feral | Kapa'a#12 | East | E |
| SRR1772239 | ugc_586_11 | Feral | Kapa'a#13 | East | E |
| SRR1772393 | ugc_586_12 | Feral | Kapa'a#14 | East | E |
| SRR1772399 | ugc_586_13 | Feral | Haena#1 | North | D |
| SRR1772981 | ugc_586_14 | Feral | Princeville_West#1 | North | E |
| SRR1773433 | ugc_586_15 | Feral | Kilauea#1 | North | E |
| SRR1773595 | ugc_586_17 | Feral | Waimea_Canyon_Road#1 | West | E |
| SRR1776921 | ugc_586_18 | Feral | Kealia#1 | East | E |
| SRR1776935 | ugc_586_19 | Feral | Kealia#2 | East | E |
| SRR1777197 | ugc_586_20 | Feral | Waimea#1 | West | E |
| SRR1778153 | ugc_586_21 | Feral | Waimea#2 | West | E |
| SRR1778284 | ugc_586_22 | Feral | Princeville#1 | North | E |
| SRR1781573 | ugc_586_23 | Feral | Princeville#2 | North | E |
| SRR1781812 | ugc_586_25 | Feral | K'ee#1 | North | E |
| SRR1783840 | ugc_610 | Wild | Laboratory population |  |  |
| SRR035376 | Layers-WLB | Domestic |  |  |  |
| SRR035381  SRR035382 | Broiler-BL | Domestic |  |  |  |
| SRR035379  SRR035380 | Broiler-BH | Domestic |  |  |  |
| SRR035388 | Layers-RIR | Domestic |  |  |  |
| SRR035383  SRR035384 | RJF | Domestic |  |  |  |
| SRR035375  SRR035389  SRR035390 | Layers-WLA | Domestic |  |  |  |
| SRR035385 | Layers-OS | Domestic |  |  |  |
| SRR035377  SRR035378 | Broilers-CB1 | Domestic |  |  |  |
| SRR035387 | Broilers-CB2 | Domestic |  |  |  |

Supplementary Table 2: Weighted Fst estimates between fineSTRUCTURE inferred clusters.

|  | Cluster1 | Cluster2 | Cluster3 | Cluster4 | Cluster5 | Cluster6 | Cluster7 | Cluster8 |
| --- | --- | --- | --- | --- | --- | --- | --- | --- |
| Cluster1 |  |  |  |  |  |  |  |  |
| Cluster2 | 0.1211 |  |  |  |  |  |  |  |
| Cluster3 | 0.1930 | 0.3291 |  |  |  |  |  |  |
| Cluster4 | 0.1258 | 0.2273 | 0.0734 |  |  |  |  |  |
| Cluster5 | 0.1151 | 0.2327 | 0.0607 | 0.0193 |  |  |  |  |
| Cluster6 | 0.1473 | 0.2904 | 0.0843 | 0.0218 | 0.0060 |  |  |  |
| Cluster7 | 0.2018 | 0.3389 | 0.2045 | 0.0852 | 0.0921 | 0.1262 |  |  |
| Cluster8 | 0.1171 | 0.2824 | 0.1436 | 0.0570 | 0.0556 | 0.0694 | 0.1836 |  |
| RFJ | 0.2596 | 0.4480 | 0.2420 | 0.1054 | 0.1158 | 0.0745 | 0.3322 | 0.2183 |

**Supplementary Table 3: Inferred date of admixture calculated by Globetrotter specifying Nullind0. The date of the admixture event for each cluster (Target) is given in generations (Gen.1date) along with the 95% confidence interval for the number of generations calculated by bootstrapping (Gen.1date.boots). The proportion and the best surrogate for the admixing source (Prop.source1, Best.source1 and Prop.source2, Best.source2) are provided. Source 1 is the major contributor while source 2 is the minor contributor.**

| **Target** | **Gen.1date** | **Gen.1date.boots** | | | **Prop.source1** | **Best.source1** | | **Prop.source2** | | **Best.source2** | |
| --- | --- | --- | --- | --- | --- | --- | --- | --- | --- | --- | --- |
| **Cluster1** | **8.96** | **(6.39-10.97)** | | | **0.57** | **Cluster2** | | **0.43** | | **Cluster3** | |
| Cluster2 | No admixture detected | | | | | | | | | | |
| Cluster3 | 39.89 | | (33.26-46.25) | 0.50 | | | Cluster6 | | 0.50 | | RJF |
| Cluster4 | 40.57 | | (31.36-52.16) | 0.50 | | | Cluster6 | | 0.50 | | Cluster5 |
| Cluster5 | 28.67 | | (23.80-36.29) | 0.79 | | | Cluster4 | | 0.21 | | Cluster2 |
| Cluster6 | 34.45 | | (27.58-39.94) | 0.79 | | | Cluster3 | | 0.21 | | Cluster2 |
| Cluster7 | 26.77 | | (21.16-35.74) | 0.80 | | | Cluster4 | | 0.20 | | Cluster2 |
| Cluster8 | 8.52 | | (5.36-10.95) | 0.74 | | | Cluster4 | | 0.26 | | Cluster2 |
| RJF | 42.25 | | (42.25-42.25) | 0.88 | | | Cluster4 | | 0.12 | | Cluster2 |
